# Factors associated with *Klebsiella pneumoniae* colonisation in adults hospitalised in Johannesburg, South Africa

**DOI:** 10.4102/sajid.v41i1.804

**Published:** 2026-05-05

**Authors:** Denasha L. Reddy, Ziyaad Dangor, Lyle Murray, Jacob M. Tsitsi, Jeremy S. Nel, Trusha Nana, Jeannette Wadula, Rispah Chomba, Sinenhlanhla Ndzabandzaba, Vicky L. Baillie, Courtney P. Olwagen, Shabir A. Madhi

**Affiliations:** 1 South African Medical Research Council Vaccines and Infectious Diseases Analytics Research Unit, Faculty of Health Sciences, University of the Witwatersrand, Johannesburg, South Africa; 2 Department of Internal Medicine, Faculty of Health Sciences, University of the Witwatersrand, Johannesburg, South Africa; 3 Division of Infectious Diseases, Department of Internal Medicine, Charlotte Maxeke Johannesburg Academic Hospital, Johannesburg, South Africa; 4 Division of Infectious Diseases, Department of Internal Medicine, Chris Hani Baragwanath Academic Hospital, Johannesburg, South Africa; 5 Division of Infectious Diseases, Department of Internal Medicine, Helen Joseph Hospital, Johannesburg, South Africa; 6 Department of Clinical Microbiology and Infectious Diseases, School of Pathology, Faculty of Health Sciences, University of the Witwatersrand, Johannesburg, South Africa; 7 Department of Microbiology, National Health Laboratory Service, Johannesburg, South Africa; 8 Wits Infectious Diseases and Oncology Research Institute, Faculty of Health Sciences, University of the Witwatersrand, Johannesburg, South Africa

**Keywords:** *Klebsiella pneumoniae*, invasive disease, gastrointestinal colonisation, infection prevention and control, presumed healthcare-associated infection, central venous catheter

## Abstract

**Background:**

There is a paucity of data on gastrointestinal colonisation with *Klebsiella pneumoniae* (KPn) in African adults, although it is a known risk factor for developing KPn invasive disease (KPn-ID).

**Objectives:**

We investigated the risk factors for KPn gastrointestinal colonisation among a cohort of hospitalised adults without KPn-ID in South Africa, and described their clinical outcomes.

**Method:**

A cohort of hospitalised adults without KPn-ID was enrolled between 15 May 2023 to 14 May 2024 across three hospitals in Johannesburg, South Africa. Study participants had rectal swab cultures performed once, at enrolment, to determine the presence or absence of KPn gastrointestinal colonisation, and were followed up until in-hospital death or discharge.

**Results:**

Among the cohort of hospitalised adults without KPn-ID (*n* = 651), the rate of KPn gastrointestinal colonisation was 34.4% (224/651). Risk factors for KPn colonisation were peptic ulcer disease (adjusted odds ratio [aOR] 7.75; 95% confidence interval [CI]: 1.34-44.78), a central venous catheter (aOR 3.07; 95% CI: 1.00-9.40), a presumed healthcare-associated infection (pHAI) (aOR 3.13; 95% CI: 1.06-9.28), and length of stay more than seven days prior to enrolment (aOR 1.84; 95% CI: 1.23-2.75). There was no difference in in-hospital case fatality risk (CFR) between participants with (3.1%; 7/224) and without (3.0%; 13/427, *p* = 0.955) KPn colonisation.

**Conclusion:**

KPn gastrointestinal colonisation rates among hospitalised adults without KPn-ID were higher than that of high-income settings and also varied between study sites. Length of hospital admission prior to swabbing and pHAI were associated with KPn colonisation, suggesting that infection prevention control measures play a significant role in KPn colonisation.

**Contribution:**

Large prospective cohort studies and community surveillance studies are required in settings such as ours to further investigate KPn colonisation dynamics and explore preventive strategies against KPn-ID.

## Introduction

The rise in *Klebsiella pneumoniae* (KPn) antimicrobial resistance (AMR) has contributed to high mortality rates from KPn-invasive disease (KPn-ID), including bloodstream infections (BSIs).^1^ The highest modelled rates of AMR burden in 2019 were in sub-Saharan Africa, with an estimated all-age regional death rate attributable to AMR at 27.3 deaths per 100 000,^2^ including 50 000 deaths attributable to KPn.^3^

*Klebsiella pneumoniae* is ubiquitous in the environment and can be present as a commensal in the gastrointestinal tract and nasopharynx of humans.^4^ Gastrointestinal colonisation represents a major reservoir for KPn^5,6^ and is a risk factor for KPn-ID,^7^ with four-fold higher odds of KPn-ID in colonised compared with uncolonised hospitalised individuals.^8^ The higher risk of KPn-ID associated with colonisation is particularly notable in intensive care units (ICUs), where individuals with KPn colonisation have been shown to have a 6.9 times higher odds of KPn-ID compared with those not colonised with KPn.^9^ The pathogenesis of progression from asymptomatic KPn gastrointestinal colonisation to invasive disease is not fully understood,^5,6,8^ although bacterial density of colonising strains and compromised host immunity have been postulated as risk factors.^5,10^

There is a paucity of data on the prevalence of colonisation with antimicrobial-susceptible or ‘wild-type’ KPn in hospitalised adults in Africa. A systematic review and meta-analysis of gut mucosal colonisation with extended-spectrum beta-lactamase (ESBL) producing *Enterobacterales* in sub-Saharan Africa (of which 60.0% of participants were adults) reported a pooled community colonisation prevalence of 18.0% (95% confidence interval [CI]: 12.0% – 28.0%).^11^ Furthermore, the prevalence of colonisation at hospital admission was 32.0% (95% CI: 24.0% – 41.0%) compared with 55.0% (95% CI: 49.0% – 60.0%) in individuals hospitalised for more than 24 h.^11^ A systematic review of carbapenem-resistant *Enterobacterales* (CRE) infections and colonisation in mainly African hospital settings (92.9%), identified *Klebsiella* species as the most prevalent CRE pathogen in most invasive and colonising isolates (72.2%; *n* = 11315/15666).^12^ Furthermore, previous antibiotic use (three out of three studies) and prior hospitalisation (two out of three studies) were the most common risk factors for colonisation with CRE.^12^ Another systematic review and meta-analysis reported a pooled global prevalence of carbapenem-resistant KPn (CRKp) colonisation in hospital and community settings of 5.4% (95% CI: 3.7% – 7.4%), with a regional African prevalence of 14.3% (95% CI: 9.48% – 19.97%) based on data from two African studies.^13^

We investigated the risk factors for KPn gastrointestinal colonisation amongst hospitalised adults without KPn-ID in Johannesburg, South Africa. Furthermore, we compared clinical outcomes in colonised versus uncolonised participants. The findings on the ‘clinical and microbiological epidemiology of KPn-ID’ of the participants with KPn-ID have been previously reported.^14^

## Research methods and design

### Study design and study population

We undertook a prospective observational study from 15 May 2023 to 14 May 2024 across three academic hospitals in Johannesburg, South Africa, with study sites as previously described^14^: ‘Chris Hani Baragwanath Academic Hospital (CHBAH), Charlotte Maxeke Johannesburg Academic Hospital (CMJAH) and Helen Joseph Hospital (HJH)’. The largest hospital, CHBAH, is located in Soweto and has a bed capacity of approximately 3200.^14,15^ Chris Hani Baragwanath Academic Hospital serves a population of over 1.9 million from southern Johannesburg.^14^ The second largest hospital, CMJAH, has a bed capacity of 1088, and serves the central Johannesburg region,^14,16^ and HJH is located in Auckland Park, Johannesburg and serves the population from Region B of the Johannesburg Municipality with a bed capacity of 636.^14,17^ The main study was undertaken to investigate the ‘clinical and microbiological epidemiology of KPn-ID’ in our setting.^14^ Adults with KPn-ID were identified through daily laboratory-based surveillance of blood and cerebrospinal fluid (CSF) cultures at the National Health Laboratory Service (NHLS) microbiology laboratories serving each hospital. The main rationale for enrolling a group of hospitalised adults without KPn-ID that were matched to cases was to ultimately compare genomic variables in the invasive and colonising isolates (an invasiveness index), while controlling for potential confounding clinical factors. The clinical data presented here are limited to the cohort of hospitalised adults without KPn-ID. Two hospitalised adults without KPn-ID were enrolled for each KPn-ID case that was alive at the time of enrolment. The hospitalised adults without KPn-ID were matched to cases on the hospital site, age category (18–35 years of age, 36–50 years of age, 51–65 years of age, > 65 years of age) and duration of hospitalisation (< 7 days in hospital, greater than or equal to 7 days in hospital). A single rectal swab was obtained from hospitalised adults without KPn-ID at the time of enrolment to assess for KPn gastrointestinal colonisation. Participants who subsequently developed KPn-ID during hospital admission were excluded and considered as cases. The only other exclusion criterion in the study was refusal of consent to participate.

### Microbiological identification

Traditional microbiology culture methods were performed for the identification of KPn, and confirmation done with Analytical Profile Index (API) (bioMereux, France) 20E. At the University of the Witwatersrand-Vaccines and Infectious Diseases Analytics Research Unit (Wits-VIDA) laboratory, rectal swab samples were transferred to brain heart infusion (BHI) broth prior to being vortexed for 60 s to release microorganisms into the nutrient broth. The swabs were then incubated in the BHI broth for 24 h at 37 °C.

Following enrichment, 50 uL of the BHI broth was inoculated onto Colorex Orientation Agar plates (Media Mage, South Africa) and then incubated at 37 °C for up to 48 h. Following growth, colonies were identified by morphology, as well as a chromogenic colour change, and presumptive KPn identification was confirmed on isolates with API (bioMereux, France) 20E (for Gram-negative bacteria). Phenotypic antimicrobial susceptibility testing (AST) was not performed on KPn isolated from rectal swabs.

### Record review

As per the main study, hospitalised adults without KPn-ID were approached to consent for study participation.^14^ Clinical and laboratory information was extracted from clinical records and entered into a password-protected electronic database (REDCap).^14^ Clinical data included a history of comorbidities, including human immunodeficiency virus (HIV) co-infection and diabetes, and risk factors for presumed healthcare-associated infections (pHAIs) such as recent surgery, presence of a central venous catheter (CVC) and urethral catheterisation. At the time of enrolment, participants’ sterile site culture results (blood and CSF) from the current admission were also reviewed for non-KPn organisms.

Participants were classified as having pHAIs if the sample from which a non-KPn organism was cultured was obtained ≥ 48 h after admission to the hospital, or if there was previous contact with a healthcare service in the preceding 2 weeks. Presumed community-associated infections (pCAIs) were defined as a non-KPn organism cultured from a sample obtained < 48 h after admission, and no record of previous contact with a healthcare service in the preceding 2 weeks. The quick Sepsis Related Organ Failure Assessment (qSOFA) score was used to compare the severity of illness in the colonised and uncolonised participants. The qSOFA score uses three criteria, assigning one point for low blood pressure (systolic blood pressure [SBP] ≤ 100 mmHg), high respiratory rate (≥ 22 breaths per minute), or altered mentation (Glasgow coma scale < 15). The Charlson Comorbidity Index (CCI) was calculated by extracting information on comorbidities from the clinical records of all participants at enrolment.^14^ The Barthel Index of Activities of Daily Living was used to determine the functional status of participants at two separate time points: at enrolment (to determine the baseline level of functioning before admission) and telephonically at 90-day post-discharge. As per the main study, participants were followed up until discharge from the hospital or in-hospital death.^14^ A telephonic follow-up was done at 90 days after discharge to determine the outcomes of individuals who had been discharged from hospital, either directly or via their caregivers, to determine 90-day outcomes (readmission, survival or death) in addition to performing the repeat Barthel Index.^14^

### Statistical analysis

Continuous variables were described using medians and interquartile ranges (IQRs). Categorical variables were summarised using frequencies and percentages. To test for significant differences between two groups (colonised and uncolonised participants), the chi-squared test was used for categorical variables, while the independent *t*-test was used for continuous variables. Logistic regression was used to determine risk factors for gastrointestinal colonisation amongst hospitalised adults without KPn-ID. Adjusted odds ratios [aORs] and 95% CIs for gastrointestinal colonisation were reported. Variables included in the multivariable logistic regression were variables in which the unadjusted *p*-value was < 0.1. Statistical analysis was performed using Stata/Standard Edition (SE) version 19.5SE.0 (StataCorp, College Station, Texas, United States [US]). A *p*-value < 0.05 was considered statistically significant.

### Ethical considerations

Ethical clearance to conduct this study was obtained from the Human Research Ethics Committee (HREC), University of the Witwatersrand (No. M220960), and registered on the South African National Health Research Database (reference number: GP202209026). Written informed consent was obtained for all participants enrolled during their hospital admission.

## Results

**FIGURE 1:**
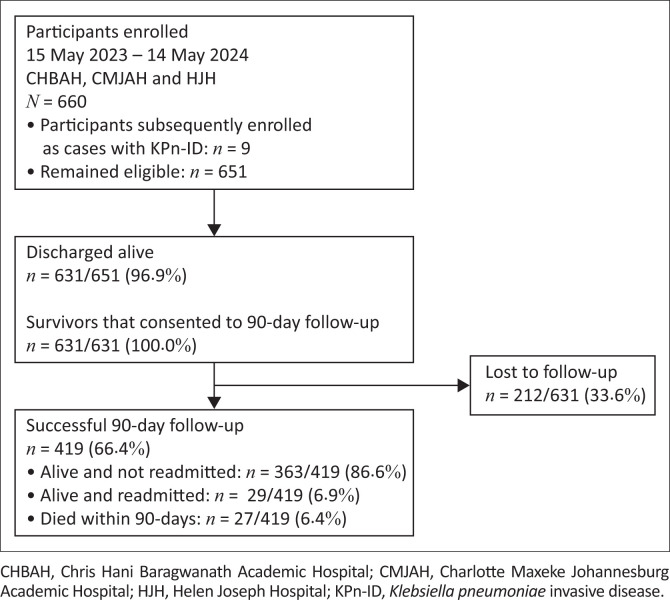
Consort diagram.

Six hundred and sixty participants without KPn-ID were enrolled. Nine individuals subsequently developed KPn-ID, including three with and six without KPn rectal colonisation at the time of enrolment as controls; Figure 1. All nine were subsequently re-enrolled as cases in the main cohort investigating KPn-ID,^14^ and were excluded from further analysis of colonisation risk factors and outcomes. The KPn-ID episode occurred at 25 days, 44 days and 134 days after having been enrolled as controls in those who were colonised by KPn; and at 9 days, 15 days, 20 days, 21 days, 68 days and 87 days after enrolment as controls in the six who were not colonised.

There were no differences in the demographic characteristics of gender, age and race between participants with and without KPn colonisation (Table 1). Rates of colonisation varied depending on the hospital study site (Table 1). Among the cohort of hospitalised adults without KPn-ID (*n* = 651), the rate of KPn gastrointestinal colonisation was 34.4% (95% CI: 30.4% – 37.6%; *n* = 224/651) overall, including 38.1% (95% CI: 33.1% – 42.9%; *n* = 143/375) at CHBAH, 29.3% (95% CI: 23.3% – 34.7%; *n* = 72/246) at CMJAH, and 30.0% (95% CI: 13.6% – 46.4%; *n* = 9/30) at HJH (Table 2). There was a lower odd of being colonised with KPn in participants enrolled at CMJAH (29.3%, *n* = 72/246) compared with those enrolled at CHBAH (38.1%, *n* = 143/375; aOR 0.68; 95% CI: 0.47–0.98) (Table 2).

**TABLE 1:** Demographic and clinical characteristics of colonised and uncolonised participants at enrolment.

Variable	Controls colonised with KPn (*n* = 224)				Controls not colonised with KPn (*n* = 427)				*p*-value*
	*n*	%	Median	IQR	*n*	%	Median	IQR	
Male sex	49	21.9	-	-	98	23.0	-	-	0.755
Age (years)	-	-	46	35.0–58.0	-	-	45	34.0–60.0	0.717
**Age categories (years)**									
≤ 18–35	61	27.2	-	-	121	28.3	-	-	0.765
36–50	69	30.8	-	-	138	32.3	-	-	0.693
51–65	69	30.8	-	-	105	24.6	-	-	0.089
> 65	25	11.2	-	-	63	14.8	-	-	0.203
**Race**									
Black African people	200	89.3	-	-	362	84.8	-	-	0.112
White people	10	4.5	-	-	36	8.4	-	-	0.061
Mixed race people	11	4.9	-	-	25	5.9	-	-	0.617
Indian descent people	3	1.3	-	-	4	0.9	-	-	0.636
**Hospital**									
CHBAH	143	63.8	-	-	232	54.3	-	-	0.020
CMJAH	72	32.1	-	-	174	40.7	-	-	0.031
HJH	9	4.0	-	-	21	4.9	-	-	0.603
**Area of admission at the time of sample collection**									
Intensive care unit	3	1.3	-	-	3	0.7	-	-	0.419
High care unit	0	0.0	-	-	0	0.0	-	-	NA
Medical ward	115	51.3	-	-	208	48.7	-	-	0.524
Surgical ward	81	36.2	-	-	147	34.4	-	-	0.659
Oncology unit	8	3.6	-	-	21	4.9	-	-	0.429
Urology	3	1.3	-	-	2	0.5	-	-	0.227
Obstetrics and Gynaecology	9	4.0	-	-	32	7.5	-	-	0.083
Transplant unit (solid organ)	0	0.0	-	-	0	0.0	-	-	NA
Other†	5	2.2	-	-	14	3.3	-	-	0.451
Previous hospitalisation within 14 days	19	8.5	-	-	37	8.7	-	-	0.937
Received antibiotics before admission	3	1.3	-	-	7	1.6	-	-	0.762
Received antibiotics during admission	128	57.1			217	50.8			0.125
Presence of comorbidities	165	73.7	-	-	312	73.1	-	-	0.871
Charlson Comorbidity Index	-	-	4	1.0–7.0	-	-	4	1.0–7.0	0.548
**Comorbid conditions**									
HIV	88	39.3	-	-	156	36.5	-	-	0.491
Diabetes mellitus	40	17.9	-	-	58	13.6	-	-	0.147
Moderate-to-severe chronic kidney disease	8	3.6	-	-	28	6.6	-	-	0.113
Solid tumour (non-metastatic)	25	11.2	-	-	63	14.8	-	-	0.203
Cerebrovascular accident	2	0.9	-	-	10	2.3	-	-	0.192
Leukaemia	0	0.0	-	-	1	0.2	-	-	0.469
Solid tumour (Metastatic)	13	5.8	-	-	23	5.4	-	-	0.825
Hemiplegia	1	0.4	-	-	3	0.7	-	-	0.691
Congestive cardiac failure	28	12.5	-	-	39	9.1	-	-	0.179
Lymphoma	4	1.8	-	-	10	2.3	-	-	0.642
Liver disease	10	4.5	-	-	8	1.9	-	-	0.055
Peptic ulcer disease	5	2.2	-	-	2	0.5	-	-	0.038
Connective tissue disease	11	4.9	-	-	15	3.5	-	-	0.387
Chronic obstructive pulmonary disease	14	6.3	-	-	22	5.2	-	-	0.560
Peripheral vascular disease	2	0.9	-	-	9	2.1	-	-	0.253
Myocardial infarction	3	1.3	-	-	8	1.9	-	-	0.615
Dementia	0	0.0	-	-	1	0.2	-	-	0.469
**CD4+ T helper cells band (cells/uL) in people living with human immunodeficiency virus (*n* = 244)**									
< 100	18	20.5	-	-	20	12.8	-	-	0.114
101–200	9	10.2	-	-	21	13.5	-	-	0.460
201–500	34	38.6	-	-	47	30.1	-	-	0.175
> 500	18	20.5	-	-	46	29.5	-	-	0.124
Unknown and/or not recorded	9	10.2	-	-	22	14.1	-	-	0.383
**Viral load band (copies/mL) in people living with human immunodeficiency virus (*n* = 244)**									
≤ 50	38	43.2	-	-	77	49.4	-	-	0.353
51–999	12	13.6	-	-	18	11.5	-	-	0.632
1000–9999	4	4.5	-	-	7	4.5	-	-	0.983
≥ 10 000	21	23.9	-	-	32	20.5	-	-	0.542
Unknown/ not recorded	13	14.8	-	-	22	14.1	-	-	0.886
On antiretroviral therapy (ART) (*n* = 244)	75	85.2	-	-	135	86.5	-	-	0.683
Recent surgery or intervention	80	35.7	-	-	144	33.7	-	-	0.612
Presence of central venous catheter	8	3.6	-	-	6	1.4	-	-	0.070
Presence of urethral catheter	10	4.5	-	-	16	3.7	-	-	0.657
**qSOFA score** ‡									
0	206	92.0	-	-	393	92.0	-	-	0.974
1	17	7.6	-	-	34	8.0	-	-	0.866
2	1	0.4	-	-	0	0.0	-	-	0.167
3	0	0.0	-	-	0	0.0	-	-	NA
Length of stay between admission and enrolment (rectal swab) (days)	-	-	10	7–16	-	-	8	6.0–17.0	0.002
Length of stay > 7 days prior to enrolment (rectal swab)	181	80.8	-	-	299	70.0	-	-	0.003
**Infection status**									
No infection	168	75.0	-	-	335	78.5	-	-	0.318
Community-associated infection	47	21.0	-	-	86	20.1	-	-	0.800
Healthcare-associated infection	9	4.0	-	-	6	1.4	-	-	0.035

Note: Controls colonised with KPn – CD4+ T helper cells band (cells/uL): *n* = 88, Viral load band (copies/mL) in people living with human immunodeficiency virus: *n* = 88. Controls not colonised with KPn – CD4+ T helper cells band (cells/uL): *n* = 156, Viral load band (copies/mL) in people living with human immunodeficiency virus: *n* = 156.

KPn, *Klebsiella pneumoniae*; IQR, interquartile range; NA, not applicable; CHBAH, Chris Hani Baragwanath Academic Hospital; CMJAH, Charlotte Maxeke Johannesburg Academic Hospital; HJH, Helen Joseph Hospital; ART, antiretroviral therapy; qSOFA, quick Sepsis Related Organ Failure Assessment; CD4, cluster of differentiation 4.

* , *p*-value resulting from chi-squared test or standard *t*-test comparison of variables in colonised versus uncolonised group. The chi-squared test or standard *t*-test was not done where the values were the same in each category.

† , Other areas of admission included neurology, neurosurgery, orthopaedic surgery, otorhinolaryngology and maxillofacial surgery wards.

‡ , Quick Sepsis Related Organ Failure Assessment (qSOFA) score uses three criteria, assigning one point for low blood pressure (SBP ≤ 100 mmHg), high respiratory rate (≥ 22 breaths per minute), or altered mentation (Glasgow coma scale < 15).

**TABLE 2:** Univariate and multivariate logistic regression risk factor analysis for *Klebsiella pneumoniae* gastrointestinal colonisation among participants.

Variable	Controls colonised with KPn (*n* = 224)		Controls not colonised with KPn (*n* = 427)		Unadjusted			Adjusted		
	*n*	%	*n*	%	OR	95% CI	*p*-value	OR	95% CI	*p*-value
**Sex**										
Male	49	21.9	98	23.0	0.94	0.64–1.39	0.755	ND	ND	ND
Female	175	78.1	329	77.0	Ref	Ref	Ref	NA	NA	NA
**Age categories (years)**										
18–35	61	27.2	121	28.3	Ref	Ref	Ref	NA	NA	NA
36–50	69	30.8	138	32.3	0.99	0.65–1.51	0.970	ND	ND	ND
51–65	69	30.8	105	24.6	1.30	0.85–2.01	0.230	ND	ND	ND
> 65	25	11.2	63	14.8	0.79	0.45–1.37	0.399	ND	ND	ND
**Race**										
Black African people	200	89.3	362	84.8	Ref	Ref	Ref	NA	NA	NA
White people	10	4.5	36	8.4	0.50	0.24–1.03	0.062	0.47	0.21–1.02	0.057
Mixed race people	11	4.9	25	5.9	0.80	0.38–1.65	0.541	0.85	0.40–1.78	0.664
Indian descent people	3	1.3	4	0.9	1.36	0.30–6.13	0.691	1.12	0.24–5.29	0.883
**Hospital†**										
CHBAH (*n* = 375)	143	38.1	232	61.9	Ref	Ref	Ref	NA	NA	NA
CMJAH (*n* = 246)	72	29.3	174	70.7	0.67	0.48–0.95	0.023	0.68	0.47–0.98	0.039
HJH (*n* = 30)	9	30.0	21	70.0	0.70	0.31–1.56	0.378	0.71	0.30–1.65	0.422
**Area of admission during sample collection**										
Intensive care unit	3	1.3	3	0.7	1.81	0.36–9.11	0.472	ND	ND	ND
Medical ward	115	51.3	208	48.7	Ref	Ref	Ref	NA	NA	NA
Surgical ward	81	36.2	147	34.4	1.00	0.70–1.42	0.985	ND	ND	ND
Oncology	8	3.6	21	4.9	0.69	0.30–1.60	0.388	ND	ND	ND
Urology	3	1.3	2	0.5	2.71	0.45–16.47	0.278	ND	ND	ND
Obstetrics and Gynaecology	9	4.0	32	7.5	0.51	0.23–1.10	0.087	ND	ND	ND
Other	5	2.2	14	3.3	0.65	0.23–1.84	0.413	ND	ND	ND
**Recent admission**										
No	205	91.5	390	91.3	Ref	Ref	Ref	NA	NA	NA
Yes	19	8.5	37	8.7	0.98	0.55–1.74	0.937	ND	ND	ND
**Presence of comorbid conditions**										
No	59	26.3	115	26.9	Ref	Ref	Ref	NA	NA	NA
Yes	165	73.7	312	73.1	1.03	0.71–1.49	0.871	ND	ND	ND
**Living with HIV and/or AIDS**										
No	136	60.7	271	63.5	Ref	Ref	Ref	NA	NA	NA
Yes	88	39.3	156	36.5	1.12	0.81–1.57	0.491	ND	ND	ND
**Diabetes mellitus**										
No	184	82.1	369	86.4	Ref	Ref	Ref	NA	NA	NA
Yes	40	17.9	58	13.6	1.38	0.89–2.15	0.149	ND	ND	ND
**Solid tumour (non-metastatic)**										
No	199	88.8	364	85.2	Ref	Ref	Ref	NA	NA	NA
Yes	25	11.2	63	14.8	0.73	0.44–1.19	0.204	ND	ND	ND
**Congestive cardiac failure**										
No	196	87.5	388	90.9	Ref	Ref	Ref	NA	NA	NA
Yes	28	12.5	39	9.1	1.42	0.85–2.38	0.181	ND	ND	ND
**Moderate-to-severe CKD**										
No	216	96.4	399	93.4	Ref	Ref	Ref	NA	NA	NA
Yes	8	3.6	28	6.6	0.53	0.24–1.18	0.119	ND	ND	ND
**Metastatic cancer**										
No	211	94.2	404	94.6	Ref	Ref	Ref	NA	NA	NA
Yes	13	5.8	23	5.4	1.08	0.54–2.18	0.825	ND	ND	ND
**Chronic obstructive pulmonary disease**										
No	210	93.8	405	94.8	Ref	Ref	Ref	NA	NA	NA
Yes	14	6.2	22	5.2	1.23	0.62–2.45	0.561	ND	ND	ND
**Connective tissue disease**										
No	213	95.1	412	96.5	Ref	Ref	Ref	NA	NA	NA
Yes	11	4.9	15	3.5	1.42	0.64–3.14	0.389	ND	ND	ND
**Liver disease**										
No	214	95.5	419	98.1	Ref	Ref	Ref	NA	NA	NA
Yes	10	4.5	8	1.9	2.44	0.95–6.29	0.063	2.63	0.99–7.03	0.053
**Peptic ulcer disease**										
No	219	97.8	425	99.5	Ref	Ref	Ref	NA	NA	NA
Yes	5	2.2	2	0.5	4.85	0.93–25.21	0.060	7.75	1.34–44.78	0.022
**Recent Surgery**										
No	144	64.3	283	66.3	Ref	Ref	Ref	NA	NA	NA
Yes	80	35.7	144	33.7	1.09	0.78–1.53	0.612	ND	ND	ND
**Presence of central venous catheter**										
No	216	96.4	421	98.6	Ref	Ref	Ref	NA	NA	NA
Yes	8	3.6	6	1.4	2.60	0.89–7.59	0.081	3.07	1.00–9.40	0.049
**Presence of urethral catheter**										
No	214	95.5	411	96.3	Ref	Ref	Ref	NA	NA	NA
Yes	10	4.5	16	3.7	1.20	0.54–2.69	0.657	ND	ND	ND
**qSOFA score**										
0	206	92.0	393	92.0	Ref	Ref	Ref	NA	NA	NA
1	17	7.6	34	8.0	0.95	0.52–1.75	0.879	ND	ND	ND
2	1	0.4	0	0.0	ND	ND	ND	ND	ND	ND
**Infection status during admission (non-KPn-ID)**										
No infection	168	75.0	335	78.5	Ref	Ref	Ref	NA	NA	NA
Community-acquired	47	21.0	86	20.1	1.09	0.73–1.63	0.674	1.10	0.73–1.66	0.652
Healthcare-associated	9	4.0	6	1.4	2.99	1.05–8.54	0.041	3.13	1.06–9.28	0.039
**Exposure to antimicrobials during admission**										
No	96	42.9	210	49.2	Ref	Ref	Ref	NA	NA	NA
Yes	128	57.1	217	50.8	1.29	0.93–1.79	0.125	ND	ND	ND
**Length of stay more than 7 days prior to enrolment (rectal swab)**										
No	43	19.2	128	30.0	Ref	Ref	Ref	NA	NA	NA
Yes	181	80.8	299	70.0	1.80	1.22–2.67	0.003	1.84	1.23–2.75	0.003

OR, odds ratio; NA, not applicable; ND, not done; KPn, *Klebsiella pneumoniae*; KPn-ID, *Klebsiella pneumoniae* invasive disease; CHBAH, Chris Hani Baragwanath Academic Hospital; CMJAH, Charlotte Maxeke Johannesburg Academic Hospital; HJH, Helen Joseph Hospital; CKD, chronic kidney disease; qSOFA, quick Sepsis Related Organ Failure Assessment; CI, confidence interval; Ref, reference.

† , Percentages in this row were calculated with the denominator as the total number of participants enrolled at that specific hospital.

There were no significant differences in the colonised versus uncolonised participants with regard to area of admission at the time of swab collection, previous hospitalisation 14 days prior to the current admission, receipt of antimicrobials prior to or during admission, the presence of comorbidities, and the CCI (Table 1).

Colonised and uncolonised participants had the same median CCI (4, IQR 1–7). There were no significant differences between the colonised and uncolonised people living with human immunodeficiency virus, even after stratification by cluster of differentiation 4 (CD4) count, viral load and exposure to antiretroviral therapy; Table 1. Although HIV, diabetes, metastatic cancer, congestive cardiac failure (CCF), connective tissue disease and chronic obstructive pulmonary disease (COPD) were more prevalent in colonised participants, the associations were not statistically significant (Table 1 and Table 2). Similarly, moderate-to-severe chronic kidney disease (CKD) and non-metastatic cancer were more frequent in uncolonised participants, but the associations were insignificant (Table 1 and Table 2). There were no significant differences between the colonised and uncolonised participants regarding exposure to recent surgery or interventions, urethral catheters, or severity of illness measured with qSOFA scores; Table 1 and Table 2. Length of stay (in days) between admission and enrolment (rectal swabbing) was longer in colonised versus uncolonised participants (median = 10, IQR 7–16 vs median = 8, IQR 6–17; *p* = 0.002); Table 1. Similarly, a length of hospital stay of more than 7 days prior to enrolment (rectal swabbing) was more likely in colonised vs uncolonised participants (80.8%; *n* = 181/224 vs 70.0%; *n* = 299/427; *p* = 0.003).

On multivariate regression analysis, risk factors for KPn colonisation were peptic ulcer disease (2.2%; *n* = 5/224 vs 0.5%; *n* = 2/427; aOR 7.75; 95% CI: 1.34–44.78), a CVC (3.6%; *n* = 8/224 vs 1.4%; *n* = 6/427; aOR 3.07; 95% CI: 1.00–9.40), a pHAI (4.0%; *n* = 9/224 vs 1.4%; *n* = 6/427; aOR 3.13; 95% CI: 1.06–9.28), and length of stay more than 7 days prior to enrolment (80.8%; *n* = 181/224 vs 70.0%; *n* = 299/427; aOR 1.84; 95% CI: 1.23–2.75); Table 2.

There was no difference in in-hospital case fatality risk (CFR) between participants with (3.1%; *n* = 7/224) and without (3.0%; *n* = 13/427, *p* = 0.955) KPn colonisation (Table 1). Of the participants, 96.9% (*n* = 631/651) were discharged alive, and a successful 90-day post-discharge follow-up was achieved in 66.4% (*n* = 419/631) (Figure 1). Participants colonised with KPn had a higher 90-day CFR compared to the uncolonised (6.5%; *n* = 14/217 vs 3.1%; *n* = 13/414, *p* = 0.051) and were more likely to be readmitted at 90-day follow-up (5.1%; *n* = 11/217 vs 4.3%; *n* = 18/414, *p* = 0.045) (Table 3). Total length of hospitalisation in days was significantly longer in colonised (median 17; IQR 11–28) compared to uncolonised (median 15; IQR 9–24) participants, *p* = 0.025; Table 3.

**TABLE 3:** Comparison of in-hospital and 90-day outcomes (colonised versus uncolonised participants).

Variable	Controls colonised with KPn and discharged alive (*n* = 217)				Controls not colonised with KPn and discharged alive (*n* = 414)				*p* *
	*n*	%	Median	IQR	*n*	%	Median	IQR	
**Length of hospitalisation**									
Length of hospitalisation from enrolment to final outcome (days)	-	-	5	1–11	-	-	4	1.0–11.0	0.512
Length of hospitalisation from admission to final outcome (days)	-	-	17	11–28	-	-	15	9.0–24.0	0.025
**In-hospital outcome**									
Demised	7	3.1	-	-	13	3.0	-	-	0.955
Discharged	217	96.9	-	-	414	97.0	-	-	0.955
**Outcomes- 90-day follow-up**									
Alive	123	56.7	-	-	269	64.9	-	-	0.045
Readmitted	11	5.1	-	-	18	4.3	-	-	-
Not readmitted	112	51.6	-	-	251	60.6	-	-	-
Lost to follow-up	80	36.9	-	-	132	31.9	-	-	0.208
Demised	14	6.5	-	-	13	3.1	-	-	0.051

KPn, *Klebsiella pneumoniae*; IQR, interquartile range.

* , *p*-value resulting from chi-squared test or standard *t*-test comparison of variables in colonised versus uncolonised group.

## Discussion

This multicentre observational study adds important evidence on risk factors associated with KPn colonisation in South Africa, a setting where the colonisation rate appears higher than in most high-income countries. We demonstrated a KPn colonisation rate amongst hospitalised adults without KPn-ID that ranged from 29.3% to 38.1% across three hospitals, substantially higher than that reported in European^7^ or Australian^9^ cohorts, suggesting a greater baseline risk in our setting.

There is a paucity of data on drug-susceptible KPn colonisation in Africa, as most high-quality studies from the region focus on AMR-related KPn colonisation and KPn-ID.^11,18,19^ The observed KPn colonisation rate in our study (34.4%) is markedly higher than rates reported in hospitalised Australian (6.0% – 19.0%)^9^ and healthy Norwegian (16.3%)^7^ adults, but lower than the 62.0% prevalence in healthy Chinese adults.^20^ A large cross-sectional study by Huynh et al.^21^ investigated KPn gastrointestinal colonisation in asymptomatic pregnant women from low-income communities in Madagascar (64.7%), Cambodia (66.4%), and Senegal (40.2%) reported an overall colonisation rate of 55.9% (*n* = 489/874).

Gastrointestinal colonisation rates also differ according to different KPn AMR profiles. A 2019 South African point-prevalence study investigating CRE gastrointestinal colonisation in hospitalised adults reported a prevalence of 0.2% (*n* = 1/439) of CRKp.^22^ Another South African study (2021) investigating CRKp colonisation via serially collected rectal swabs (days 1, 3, 7 and weekly until transfer, discharge or death) in adults admitted to ICU reported a cumulative prevalence of 16.1% (*n* = 14/97 swabs from *n* = 5/31 patients).^23^ A study from Bloemfontein, South Africa, investigating the prevalence of, and gastrointestinal colonisation with, multidrug-resistant (MDR) organisms amongst adults with CKD undergoing outpatient haemodialysis and peritoneal dialysis reported a prevalence of MDR KPn of 21.1% (*n* = 15/71).^24^ This variability in colonisation rates across different geographical regions and different institutions within the same city as demonstrated in our study, highlights the complex interaction of host, pathogen ecology, and healthcare system factors, with infection-prevention practices likely contributing to the differences.^7,19,25^

Risk factors for KPn gastrointestinal colonisation have been reported primarily in high-income settings and include ICU admission and exposure to antimicrobial therapy.^7,9,10^ While there was an increased risk for KPn colonisation associated with CVCs, we did not demonstrate an increased risk with ICU admission, surgery or urethral catheters, although this may be because of the relatively small number of critically ill participants enrolled from the ICU. In keeping with published literature, we found a length of hospital stay of more than 6 days prior to enrolment (rectal swabbing) and a pHAI to be independently associated with KPn colonisation,^10^ suggesting that hospitalisation itself drives colonisation pressure in our hospitals. In terms of comorbidities, we found peptic ulcer disease to be a significant independent risk factor for colonisation. In a community-based cross-sectional study of 2975 Norwegian adults, Raffelsberger et al.^7^ reported that the use of proton pump inhibitors (PPIs) (aOR 1.62; 95% CI: 1.18–2.22) and non-steroidal anti-inflammatory drugs (NSAIDs) within the past 6 months (aOR 1.38; 95% CI: 1.04–1.84) were independent risk factors for KPn gastrointestinal colonisation. Both PPIs and NSAIDs are associated with peptic ulcer disease treatment and aetiology, respectively, which suggests a common pathophysiological state involving gut microbiota changes that favours KPn colonisation.^7^ Huynh et al.^21^ demonstrated a higher risk of KPn colonisation following the use of antibiotics during pregnancy (in Madagascar), dry fish consumption (in Cambodia) and contact with chickens (in Senegal). These findings suggest that, in addition to the traditional risk factors associated with colonisation in hospitalised adults, food and animal exposure are likely underestimated risk factors for KPn colonisation in community settings, highlighting the need for a one-health approach.

There is scarce regional data on the clinical outcomes of KPn colonised individuals. Although gastrointestinal colonisation is widely recognised as a prerequisite for invasive disease,^7,8,9,10,26,27^ in our study, only three out of nine participants who went on to develop KPn-ID were colonised at enrolment. This finding is not unexpected as KPn gastrointestinal colonisation is a dynamic process,^10,28^ and our study participants were swabbed at only one time point. A recent systematic review and meta-analysis investigating the ‘incidence and risk factors for subsequent infections amongst rectal carriers with CRKp’ reported that multi-site colonisation (OR 6.24; 95% CI: 2.38–16.33) was an independent risk factor for subsequent CRKp disease.^29^ Although we did not find differences in in-hospital CFR amongst colonised versus uncolonised participants, we did note a trend towards poorer clinical outcomes in the colonised group with higher 90-day CFR and readmission rates.

Limitations of our study include that participants were not randomly selected but matched to invasive cases based on age group, duration of hospitalisation prior to enrolment and study site, leading to inadvertent bias in participant selection and estimation of colonisation rate. Additionally, gastrointestinal colonisation is dynamic^9^ and the fact that participants in our study were only swabbed at enrolment and from a single site (rectal swab) likely underestimated the rate of KPn gastrointestinal colonisation in our setting, and also raises the question of whether the colonising KPn isolates were acquired in hospital or represent the patient’s own gut microbiota. Also, phenotypic AST was not performed on rectal swab KPn isolates. Furthermore, approximately 33.6% of the discharged participants were not traceable for the 90-day follow-up, which may lead to an under-estimate of mortality in the study.

Our study has several important implications. The high burden of KPn colonisation in South African hospitals suggests that prevention strategies must extend beyond antimicrobial stewardship to include stricter infection-prevention practices. To accurately determine KPn gastrointestinal colonisation rates in South Africa, prospective surveillance studies should be undertaken in both community and hospital settings, with a one-health approach to investigating risk factors for KPn colonisation. Given the dynamic nature of KPn gastrointestinal colonisation, longitudinal surveillance in both community and hospitalised cohorts is essential to understand transmission pathways and to design interventions that interrupt progression from colonisation to disease.
